# Peripartum Subtotal Hysterectomy in Multifetal Gestation

**DOI:** 10.7759/cureus.65811

**Published:** 2024-07-31

**Authors:** Paidi Naga Rachana, Bharathna Reddy Chennuru, Sneha Prasad, Jayshree Kulkarni

**Affiliations:** 1 Obstetrics and Gynecology, Dr. D. Y. Patil Medical College, Hospital and Research Centre, Dr. D. Y. Patil Vidyapeeth (Deemed to be University), Pimpri, IND

**Keywords:** peripartum hysterectomy, post partum hemorrhage, near miss event, gestational age. parity, di-di twin gestation

## Abstract

Peripartum hysterectomy (PH) is usually undertaken in cases of life-threatening obstetric haemorrhage to prevent the death of the mother. Obstetric haemorrhage, a leading indication for PH, is a major cause of maternal deaths globally, particularly in regions with limited access to advanced medical care. The cause of the per vaginal bleeding was due to the patient in labour with a cervical stitch, and immediate action was taken in the form of a lower segment caesarean section. After the patient's abdominal drain is noticed with fresh blood collection, an emergency obstetric hysterectomy is done.

## Introduction

Peripartum hysterectomy (PH) is a critical surgical procedure performed as a last resort to control severe obstetric haemorrhage unresponsive to conservative treatments. Despite advancements in obstetric care, PH remains a significant cause of maternal morbidity and mortality, particularly in high-risk pregnancies [1]. Obstetric haemorrhage, the primary indication for PH, accounts for a considerable proportion of maternal deaths globally, especially in developing countries where access to advanced medical care may be limited [2].

Multiple studies have identified various risk factors associated with the need for PH, with uterine atony and morbidly adherent placentas being the most common indications. High parity, previous caesarean sections, and placenta previa are significant risk factors [3,4].

In our case study, we observed a unique dimension involving traumatic postpartum haemorrhage in a multifetal gestation, a scenario less commonly emphasized in the existing literature. This study aims to provide a comprehensive analysis of our case in the context of existing research, highlighting the complexities of managing severe obstetric haemorrhage in high-risk pregnancies involving assisted reproductive technology and multiple prior uterine surgeries. This study will add to the existing body of knowledge by improving our understanding of the risk factors and complications associated with PH, thereby informing clinical protocols and improving maternal and neonatal outcomes.

## Case presentation

A 37-year-old female, G3P2L0A1, 28.5 weeks of gestation, conceived through Introvitro fertilisation with Dichoriomic Diamniotic twins, and with a history of previous Lower segment cesserean section and cervical stitch in situ, presented with complaints of pain in abdomen since one day. She had a history of cervical cerclage due to a short cervix (Shirodkar's method using mercelene tape), metroplasty at the fundus and right lateral wall, and hysteroscopic adhesiolysis in the left lateral wall. She was also Rubella IgG positive. The patient had been taking Inj. Enoxaparin 40 mg subcutaneously once daily since embryo transfer day and Tab Ecospirin 75 mg once daily. She had been admitted three times at 2 Months of gestation due to Per vaginal bleeding with abdominal pain at an outside hospital.

Course of labour

On admission, the cervix was 2-2.5 cm dilated, and the membranes were absent. An ultrasonography (USG) obstetric scan was done for presentation and AFI (Twin A: cephalic; Twin B: transverse lie; AFI: Adequate Amniotic Fluid Index). The patient was shifted to emergency LSCS due to a previous LSCS with twin gestation in latent labour.

Post-operative course

**Figure 1:**
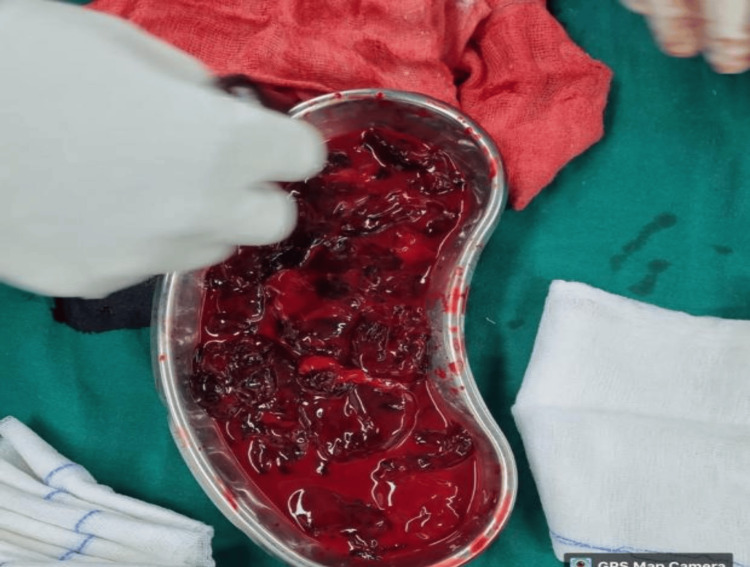
Blood clots (700 g)

**Figure 2:**
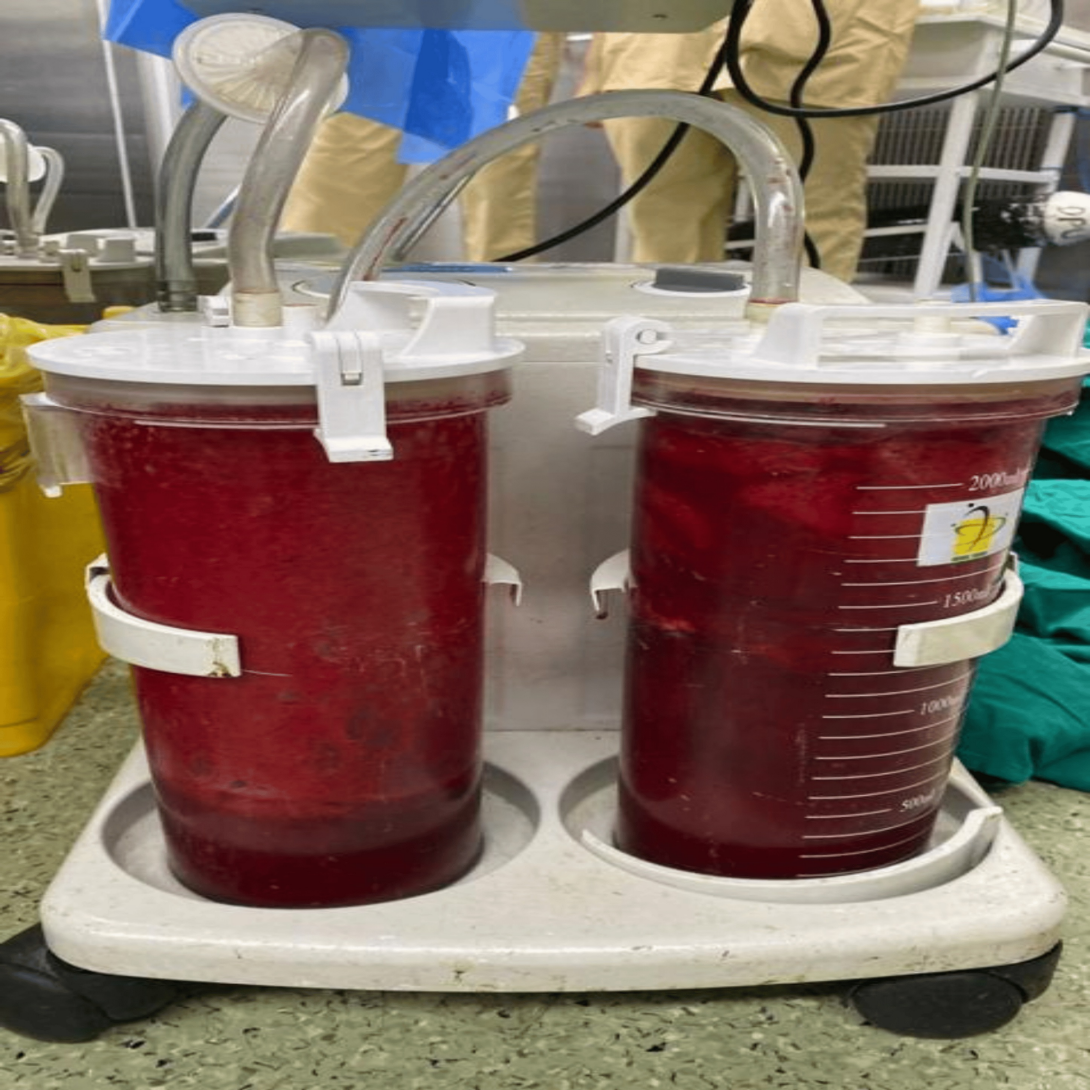
Blood collected in suction containers

**Figure 3:**
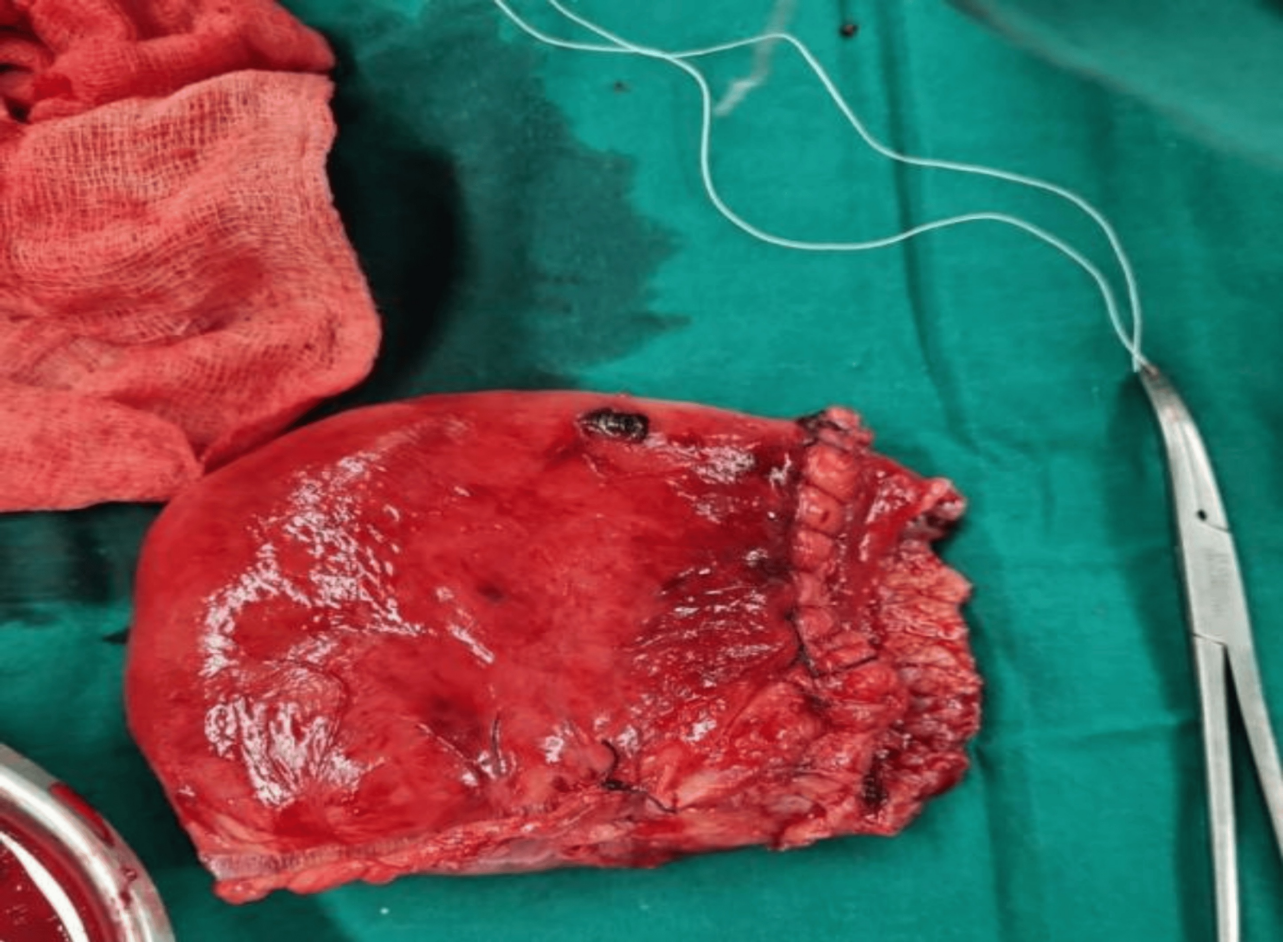
Specimen

The patient was shifted to the surgical intensive care unit (SICU) with an abdominal drain in situ and under close observation (BP: 120/70 mmHg, PR: 126 bpm, saturation: 100% on RA). The patient was on adrenaline at 2 ml/hr and received one packed cell volume (PCV) and two fresh frozen plasmas (FFP). She was then on continuous Inj. adrenaline support at 8 ml/hr (BP: 90/60 mmHg, PR: 156 bpm). Two hours after shifting to the SICU, the tone of the uterus was intermittently flabby (BP: 50/30 mmHg, PR: 158 bpm, dressing dry, minimal collection in the abdominal drain). The patient was started on Inj. vasopressin infusion at 1-2 ml/hr and adrenaline at 10 ml/hr. The uterus was still intermittently flabby, and a vaginal pack was in situ with four sponge holders on the cervix lips. At around 2:30 am, a USG abdomen pelvis was done, revealing signs of hyperechoic collection inside the uterus with clots and minimal collection in the perihepatic and perihepatic spaces. A decision was made for emergency subtotal obstetric hysterectomy due to primary postpartum haemorrhage (PPH), hypotension, tachycardia, increased requirement of inotropic support, and USG findings suggestive of clots. HB-6.3 g/dl (biological reference range: 11.6-15 g/dl). The patient was shifted to the operation theatre (OT) at 3:05 a.m. The abdomen was reopened, and 700 g of blood clots were collected (Figure 1: blood clots (700 g)). A subtotal obstetric hysterectomy was done, and blood loss of 2,500 ml was noticed (Figure 2: blood collected in suction containers). Blood and blood products transfusion was going on, and specimens (Figure 3: Specimen) were sent for HPE. The patient was shifted to the SICU with an abdominal drain and Foley's in situ for further management and observation. The patient received seven PCVs, six FFPs, and one SDP over two days.

She remained vitally stable. Drain in situ: On Inj. Piptaz, Metronidazole, and Amikacin, repeat Hb: 8.4 g/dl (biological reference range 11.6-15 g/dl). USG abdomen pelvis was done with no sign of any collection noted. The fever profile was sent and came back negative. The patient was shifted to the ward after five days. On POD5, the abdominal drain was removed, and Foley's removal was done on POD9. On POD 10, the patient started complaining of a fever. Two-hourly temperature charting was done, and a repeat CBC was sent. CSR was done on POD11, and the dressing was removed under aseptic precautions. Discharge from the suture site was noted, and wound culture and sensitivity were sent. On POD 11, the patient had a temperature of 101.1 °F. A high vaginal swab, blood culture, urine C/S, and USG abdomen and pelvis were advised. A medicine reference was given, and antibiotics were advised Inj. Augmentin, 1.2 g TDS. After two days, the patient was reviewed due to a fever associated with burning micturition. She was advised to take Inj. Tigecycline 100 mg stat, followed by 50 mg BD for five days. The USG abdomen pelvis showed collection in the pelvis at the superolateral aspect of the urinary bladder, a few collections at the suture site, and subcutaneous edoema superior to the suture line. Surgery reference advised CECT abdomen.

On POD16, the case was discussed with the head of the department (HOU). On PV examination, a collection extending to the anterior abdominal wall was revealed, and a contrast-enhanced computerised topography (CECT) was done to trace the provisional report. The CECT report showed intra-peritoneal abscesses in the pelvis, posterosuperior to the urinary bladder, and anterior to the rectum, with a possible cutaneous draining sinus tract and subcutaneous collection at the incision site. The case was informed to HOU, and a decision was made for an emergency colpotomy with a possible laparotomy. The procedure was done on February 21, post-operative day (POD17). The patient was shifted to the ward, vitally stable, with an abdominal drain and Foley's in situ. On POD2 of the emergency colpotomy, diagnostic aspiration was done, revealing a hematoma collection but no abscess. On POD3, the drain was removed. Twin A, a male child, expired on day 8 due to cardiac arrest, and Twin B, a female child, expired on day 13 due to cardiorespiratory arrest with pulmonary haemorrhage and disseminated intravascular coagulation (DIC) with multi-organ dysfunction syndrome (MODS). The patient was discharged on POD24 of the obstetric hysterectomy and POD4 of the emergency colpotomy.

## Discussion

Our case study of traumatic haemorrhage in a multifetal gestation adds a unique dimension to the existing literature, emphasising the complexities associated with IVF conception and multiple prior uterine surgeries. This discussion compares and contrasts our findings with those of previous studies, highlighting similarities and differences in the risk factors, indications, and outcomes associated with peripartum hysterectomy.

Several studies have identified common risk factors for PH, which align with those observed in our case. Yamani Zamzami noted high parity, previous caesarean sections, and placenta previa as prevalent risk factors [3]. Our case study added the elements of IVF conception and multiple prior uterine surgeries, further complicating the clinical scenario. Similarly, Maraschini et al. identified advanced maternal age, previous caesarean sections, assisted reproductive technology, and multiple pregnancies as significant risk factors for obstetric haemorrhage leading to PH, findings that are consistent with our case [5].

In terms of primary indications for PH, uterine atony and uterine rupture are frequently cited in the literature. Senturk et al. and Stella D’Arpe et al. both reported uterine atony and uterine rupture as leading causes of emergency PH [4,6]. Our case also identified uterine atony as a significant factor but uniquely highlighted traumatic postpartum haemorrhage as a primary cause. While our case primarily involved traumatic postpartum haemorrhage, placental complications also played a role, aligning with findings from Kastner et al., who noted a high incidence of placenta accreta in women with previous caesarean deliveries [7].

The maternal and neonatal outcomes associated with PH are critical aspects highlighted across studies. Both Senturk et al. and Maraschini et al. noted high maternal morbidity, including complications such as urinary bladder injury and DIC. Our case study similarly reported significant maternal morbidity, including DIC and uterine rupture. The necessity for intensive care and high transfusion rates was emphasised in both our case study and the findings of Kallianidis et al., who reported that blood transfusions and ICU admissions were necessary for a substantial proportion of patients [8]. In our case, the patient required extensive blood transfusions and ICU care, underscoring the severity of such cases.

The incidence of maternal mortality associated with delayed hospital presentations and home deliveries is another critical point. Both Senturk et al. and our case study noted high neonatal and maternal mortality rates under these circumstances [4]. Similarly, Varalakshmi et al. reported a high incidence of septicemia as a postoperative complication, which aligns with the infection and fever complications managed with antibiotics in our case [9].

Despite these similarities, there are notable differences between our case study and the existing literature. Our case involved a multifetal gestation conceived through IVF with a history of multiple uterine surgeries and a cervical stitch, leading to a more complex clinical picture. This aspect was not prominently featured in the analysis of Kastner et al. of 47 cases, which primarily focused on placenta accreta and uterine atony [7]. Additionally, while Tsolakidis et al. reported both total and subtotal hysterectomies, our case specifically involved a subtotal obstetric hysterectomy, highlighting a potentially less invasive approach that was associated with fewer complications and shorter operation times [10].

Another distinction is the specific focus on traumatic postpartum haemorrhage in our case study, which is less commonly highlighted in the literature. While studies like those by Senturk et al. and D’Arpe et al. identified uterine atony and rupture as significant factors, the emphasis on traumatic haemorrhage adds a unique dimension to our findings [4,6]. Furthermore, the high incidence of maternal and neonatal complications in our case, including the deaths of both twins due to complications, underscores the critical nature of multifetal gestation and the need for timely and appropriate intervention.

## Conclusions

This case study highlights the complexities of traumatic postpartum hemorrhage in multifetal pregnancies conceived through IVF, particularly in patients with extensive uterine surgical histories. It emphasizes the need for prompt intervention to reduce maternal morbidity and mortality, as severe complications, such as disseminated intravascular coagulation, can arise in these cases. Additionally, it identifies traumatic postpartum hemorrhage as a significant cause of peripartum hysterectomy and underscores the tragic neonatal outcomes associated with delayed intervention. Overall, this case advocates for improved risk assessment and clinical protocols to enhance care in high-risk obstetric situations.
